# The Role of Inflammatory Factors in the Pathogenesis of Gestational Diabetes Mellitus and May Be Potential Biomarkers for Its Diagnosis and Prognosis

**DOI:** 10.1155/humu/4623346

**Published:** 2025-11-17

**Authors:** Yuanyuan Guo, Xian Zheng, Jingru Jiao, Hongli Wu, Yan An

**Affiliations:** Department of Obstetrics, Affiliated Hospital of Hebei University, Baoding, Hebei Province, China

**Keywords:** gene expression, gene polymorphism, gestational diabetes mellitus, inflammatory factors

## Abstract

**Background:**

The biomarkers associated with gestational diabetes mellitus (GDM) remain incompletely understood. This article is aimed at investigating whether inflammatory factors may contribute as risk factors for GDM.

**Methods:**

The study included 160 adult patients with GDM, who were enrolled as the experimental group. Additionally, 280 healthy individuals from the same time period were selected as the control group. Cytokine expression levels were measured using a flow cytometer with fluorescence, while gene polymorphisms were analyzed through the polymerase chain reaction-restriction fragment length polymorphism (PCR-RFLP) technique. The cytokines examined included interleukin-1 (IL-1), interleukin-6 (IL-6), interleukin-10 (IL-10), tumor necrosis factor-alpha (TNF-*α*), and interferon-gamma (IFN-*γ*).

**Results:**

Significantly higher expression levels of IL-1, IL-6, IL-10, and TNF-*α* were detected in GDM patients (*p* < 0.05). Additionally, the study identified specific polymorphisms—IL-1*β* −511 C/T, IL-10 −1082 G/A, IL-6 −174 G/C, and TNF-*α* −308 G/A—that were significantly associated with an increased risk of GDM (*p* < 0.05). IL-6, TNF-*α*, and IL-1*β* levels significantly differed among genotypes of IL-6 −174 G/C, TNFA −308 G/A, and IL-1B −511 C/T, respectively (*p* < 0.01), with risk-associated alleles linked to higher cytokine expression. No significant differences were observed for IL-10 −1082 G/A or IFN-*γ* +874 A/T. These results suggest that select polymorphisms may regulate cytokine levels relevant to GDM inflammation.

**Conclusion:**

Elevated plasma levels of IL-1, IL-6, IL-10, and TNF-*α* have been observed in patients with GDM. Furthermore, polymorphisms such as IL-1*β* −511 C/T, IL-6 −174 G/C, IL-10 −1082 G/A, IFN-*γ* +874 A/T, and TNF-*α* −308 G/A show a strong correlation with an increased risk of GDM in the Han women from northern China (specifically, Hebei Province). Pregnant women with ACC haplotypes of IL-10 have a lower risk of GDM. Cytokine gene polymorphisms in IL-6, TNF-*α*, and IL-1B are associated with altered inflammatory profiles in GDM, suggesting a genetic contribution to disease-related immune dysregulation. Our study suggests that these factors hold potential as biomarkers for the diagnosis and clinical prognosis of GDM in Han women from northern China (Hebei Province).

## 1. Introduction

Gestational diabetes mellitus (GDM) is a complex metabolic disorder, and it is characterized by inadequate insulin secretion and insulin resistance. In Asia, the prevalence reaches 20.9%, while in China, it stands at 14.8% [1–4], making GDM the most common pregnancy complication. In recent years, with the improvement of detection methods and living standards, the detection rate has been continuously increasing. It is estimated that the prevalence of GDM has increased by more than 35% over the past few decades and is still on the rise [5]. Due to different diagnostic criteria, the prevalence varies in different countries and regions. It is estimated that when diagnosed with GDM using the current World Health Organization (WHO)–recognized International Association of Diabetes and Pregnancy Study Groups (IADPSG) criteria, the prevalence is approximately 6–11 times that of other diagnostic criteria. But under all diagnostic criteria, East Asia has shown the highest prevalence of GDM [6]. In addition, patients with a history of GDM have a very high risk of developing Type 2 diabetes mellitus (T2DM) within 3–6 years after the end of pregnancy [7]. Even more seriously, the offspring of untreated GDM pregnant women have more severe insulin resistance and are prone to glucose metabolism disorders [8]. However, its underlying etiology remains unclear due to its complexity. In women with a history of GDM, the presence of islet beta-cell antibodies may partially explain the condition. Genome-Wide Association Studies (GWASs) have opened new avenues for exploring the causes of GDM. Large-scale GWASs have identified many susceptibility genes for GDM that overlap with those of T2DM, such as IGF2BP2, MTNR1B, TCF7L2, INSR, IRS1, HHEX, CDKAL1, GCK, and KCNQ1, most of which are associated with insulin secretion. However, not all T2DM susceptibility loci are present in GDM, highlighting important differences between the two conditions.

Many studies suggest that inflammation-induced insulin resistance greatly contributes to GDM development. The levels of proinflammatory and anti-inflammatory factors from various sources are constantly changing, which is critical for maintaining glucose metabolism homeostasis. C-reactive protein (CRP) is the most commonly used clinical marker for measuring inflammation. In 1999 and 2009, researchers found a strong association between CRP levels and insulin sensitivity in both healthy and overweight individuals. In patients with Type 2 diabetes, CRP levels also showed a significant upward trend and were independently correlated with insulin resistance, insulin secretion, and lipid profiles. Furthermore, in patients with GDM, a prospective cohort study conducted in 2003 found that pregnant women with elevated CRP levels in the first trimester had a higher risk of developing GDM later in pregnancy, suggesting that CRP could serve as a predictor of GDM. A 2017 study in Inner Mongolia, China, also demonstrated an independent positive correlation between high-sensitivity CRP and GDM. Additionally, genetic evidence indicates that single-nucleotide polymorphisms (SNPs) in the CRP gene may increase the risk of diabetes.

When it comes to inflammatory factors, most studies focus on IL-1*β*, IL-6, IL-10, and TNF-*α*. While many believe these inflammatory markers play a detrimental role in the onset and progression of diabetes, previous research findings have been inconsistent. Some studies report increased expression of these inflammatory factors in GDM patients, while others suggest no significant change in expression levels. Moreover, the results of gene polymorphism studies on these inflammatory factors vary widely, and data from many countries and regions remain unreported. Therefore, this study is aimed at comprehensively and thoroughly investigating the expression of key inflammatory factors and the changes in their gene polymorphisms in GDM patients.

## 2. Materials and Methods

### 2.1. Study Subjects

GDM patients treated at the Affiliated Hospital of Hebei University from April 2022 to June 2024 were enrolled, comprising 160 individuals. Then, 280 healthy pregnant women were enrolled as the control group. Strict inclusion criteria were applied: Following the International Association of the Diabetes and Pregnancy Study Groups (IADPSG, 2014) guidelines, the “one-step method” for diagnosing GDM was used. This involved administering a 75-g oral glucose tolerance test (OGTT) after at least 8 h of fasting, with blood glucose levels measured at fasting, 1 h, and 2 h postglucose intake. A diagnosis of GDM was made if any of the following criteria were met: fasting blood glucose (FBG) ≥ 5.1 mmol/L, 1-h blood glucose ≥ 10.0 mmol/L, or 2-h blood glucose ≥ 8.5 mmol/L. All participants were aged 20–45 and had a singleton pregnancy. Women with preexisting diabetes, heart disease, or liver, kidney, or other related conditions were excluded. The study was approved by the Affiliated Hospital of Hebei University (Approval No. HDFYLL-KY-2024-213).

A cytokine detection kit was used to capture specific antibodies. This method utilizes flow cytometry to form a microbead array. The detection reagent was a PE-labeled (fluorescent) antibody mixture. After incubating the capture microspheres (APC), detection antibodies (PE), and the sample together, a double-antibody sandwich complex is formed.

### 2.2. Gene Polymorphism Analyses

PCR-RFLP was carried out following established protocols. Gene polymorphism analysis was performed using the ABI3730XL system, with sequencing conducted for further verification. The selection of gene loci mainly considers the following factors: First of all, these loci are located in the continuous region from the gene regulatory region to the coding region, which has been proved to have a great impact on gene function, and may be related to the occurrence and development of GDM. Secondly, the minimum allele frequency (MAF) of the Chinese Han population shown in the public gene database was ≥ 0.05, and there was linkage imbalance between them. Finally, two authors independently browse and review the Google Chrome http://hapmap.ncbi.nlm.nih.gov/cgi-perl/gbrowse/hapmap27_B36/Human genome database, load the haplomap in the Human Genome Project (HGP) into the genotype data of SNP sites, and then import it into the Haploview software. By determining the linkage disequilibrium parameter *r*^2^ = 0.8 (MAF > 0.05) and searching for tagSNPs (tagSNPs), only a few important tagSNPs in a chromosome region can be used to infer the entire genetic polymorphism pattern of the region without identifying all the loci. After comprehensive analysis of all aspects of information and research results, we selected corresponding polymorphic loci to be included in this study. Furthermore, the polymorphism of various single nucleotides of the IL-10 gene haplotype was analyzed by the Phase Program.

### 2.3. Receiver Operating Characteristic (ROC) Analysis

To assess the potential of inflammatory cytokines as diagnostic biomarkers, we performed ROC curve analysis.

For each marker, we plotted ROC curves by comparing cytokine expression values. The area under the ROC curve (AUC) was calculated. To determine the optimal diagnostic threshold for each cytokine, the Youden index (*J* = sensitivity + specificity − 1) was used. Based on this threshold, we classified individuals into predicted GDM or non-GDM categories and computed the following performance metrics: sensitivity (true positive rate), specificity (true negative rate), and accuracy (overall correct classification rate).

All ROC analyses were performed using Python 3.11, with the scikit-learn library for statistical modeling and the matplotlib package for visualization. Confidence intervals for AUC values were estimated using bootstrapping (*n* = 1000 resamples) where applicable.

### 2.4. Statistical Analysis

Hardy–Weinberg equilibrium (HWE) for genotype distributions was tested using the Chi-square test. Logistic regression analysis was applied to estimate the association between gene polymorphisms and the risk of GDM, adjusting for potential confounders. To assess the potential regulatory effects of cytokine gene polymorphisms on protein expression, we analyzed the differences in plasma cytokine levels across genotype groups for each SNP. The normality of cytokine concentration distributions was evaluated. As most variables did not follow a normal distribution, non-parametric methods were applied. Differences among genotype groups (e.g., GG, GA, and AA) were compared. Where significant differences were observed, pairwise comparisons were conducted. Box plots were generated to visualize cytokine levels across genotypes, and analysis and visualization were performed using Python (v3.11) with the SciPy and Seaborn libraries. This thesis was written with reference to the following article during the writing process (https://pubmed.ncbi.nlm.nih.gov/40000789/) [9].

## 3. Results

### 3.1. Participants

The key data are presented in Table 1, including age, gestational weeks, TG, TC, HDL-C, and LDL-C levels. The general information shows no significant differences (*p* > 0.05).

### 3.2. Cytokine Assay

### 3.3. Cytokine Expression Level

**Figure 1:**
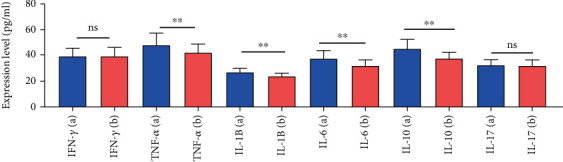
Comparison of inflammatory cytokine expression levels between GDM patients and healthy pregnant women. (a) Cytokine levels in the GDM group. (b) Cytokine levels in the control group. The expression levels of TNF-*α* cytokine expression levels were significantly higher in the GDM group compared to the control group (*p* < 0.05), while no significant differences were observed for IFN-*γ* in the GDM group compared to the control group. ⁣^ns^*p* > 0.05, ⁣^∗∗^*p* < 0.05.

Significantly higher expression levels of IL-1, IL-6, IL-10, and TNF-*α* were observed in GDM patients (*p* < 0.05) (Figure 1). However, no significant associations were found for IL-17 or IFN-*γ* (*p* > 0.05) (Figure 1).

### 3.4. Gene Polymorphism Analyses

The study found that specific polymorphisms—IL-1*β* −511 C/T, IL-10 −1082 G/A, IL-6 −174 G/C, TNF-*α* −308 G/A, and IFN-*γ* +874 A/T—were significantly associated with an increased risk of GDM (*p* < 0.05). Other loci within these genes showed no significant associations (*p* > 0.05). Detailed information can be found in Tables 2, 3, 4, 5, 6 and 7.

### 3.5. Haplotype Analysis for IL-10

All participants had one or two haplotypes of ATA, ACC, and GCC, so the genotypes were classified as ATA/ATA, ACC/ACC, ATA/ACC, ATA/GCC, ACC/GCC, and GCC/GCC. ATA/ATA was the highest in the experimental group and the control group (51.1% and 42.3%, respectively). It was followed by ATA/ACC with 27.0% and 34.6%, respectively. The ATA/GCC genotypes were 13.2% and 11.5%, respectively, accounting for third place. ACC/ACC, ACC/GCC, and GCC/GCC are rare. The haplotype frequencies of ATA, ACC, and GCC in the experimental group were 0.713, 0.195, and 0.091, respectively, and ATA was the most common haplotype. The control group had 0.645, 0.268, and 0.087. There were more ATA haplotypes in the experimental group (*p* > 0.05) and more ACC haplotypes in the control group (*p* < 0.05). The corresponding information was shown in Table 8.

### 3.6. ROC Curve Analysis Results

**Figure 2:**
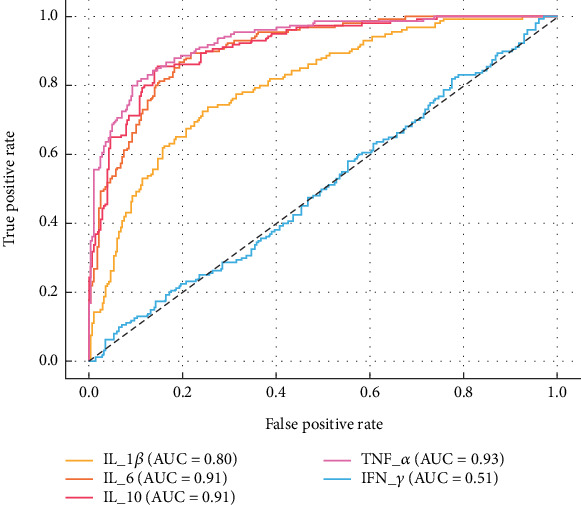
Receiver operating characteristic (ROC) curves for inflammatory cytokines in GDM diagnosis. ROC analysis was performed for IL-1*β*, IL-6, IL-10, TNF-*α*, and IFN-*γ* to assess their diagnostic performance in distinguishing GDM patients from healthy pregnant controls. The diagonal line represents the line of no discrimination (AUC = 0.5).

ROC curve analysis revealed that IL-6 demonstrated the highest diagnostic accuracy for GDM, with an AUC of 0.849, sensitivity of 79.4%, and specificity of 77.5% (Figure 2). IL-1*β* and IL-10 also showed moderate diagnostic value (AUCs = 0.822 and 0.807, respectively). TNF-*α* yielded an AUC of 0.785, while IFN-*γ* exhibited limited diagnostic performance (AUC = 0.529). These results suggest that IL-6, IL-1*β*, and IL-10 may serve as potential inflammatory biomarkers for GDM diagnosis.

### 3.7. Association Between Genotype and Cytokine Expression Levels

**Figure 3:**
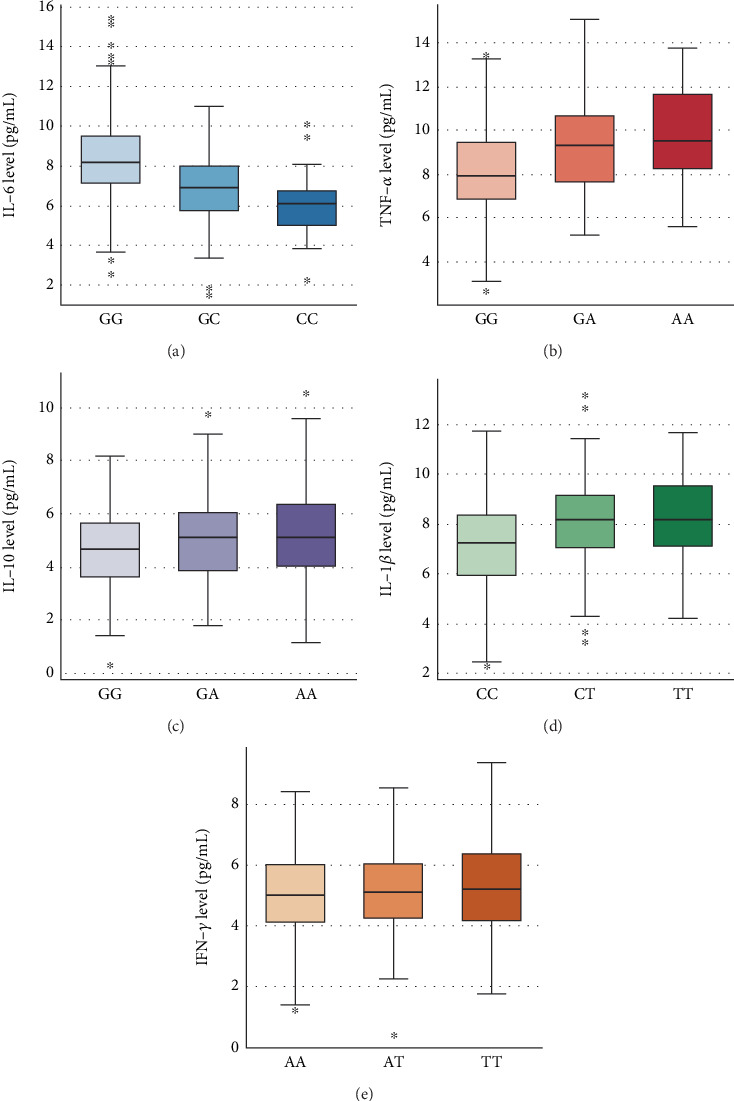
Comparison of inflammatory cytokine levels by genotype. (a) Plasma IL-6 levels among different genotypes of the IL6-174 G/C polymorphism. (b) Plasma TNF-*α* levels among different genotypes of the TNFA-308 G/A polymorphism. (c) Plasma IL-10 levels among different genotypes of the IL10-1082 G/A polymorphism. (d) Plasma IL-1*β* levels among different genotypes of the IL1B-511 C/T polymorphism. (e) Plasma IFN-*γ* levels among different genotypes of the IFN-*γ* +874 A/T polymorphism. Boxes represent the interquartile range (IQR), whiskers indicate 1.5 × IQR, and horizontal lines within boxes denote the median. Differences between genotypes were evaluated using the Kruskal–Wallis test followed by Dunn's post hoc test where applicable.

To investigate whether inflammatory cytokine gene polymorphisms influence protein expression, we compared plasma cytokine levels among genotype groups (Figures 3a, 3b, 3c, 3d, and 3e).

For the IL-6 −174 G/C polymorphism, IL-6 levels differed significantly across genotypes (*p* < 0.001; Figure 3a). The GG genotype showed the highest concentrations (median [IQR]: 8.4 [7.2–9.7] pg/mL), followed by GC (6.6 [5.4–7.5] pg/mL) and CC (5.8 [4.9–6.5] pg/mL). Post hoc analysis revealed significant differences between GG and GC (*p* = 0.004) and between GG and CC (*p* < 0.001), but not between GC and CC (*p* = 0.12).

For the TNF- *α* −308 G/A polymorphism, TNF-*α* levels varied significantly among genotypes (*p* = 0.001; Figure 3b). Individuals with the AA genotype had the highest levels (mean ± SD : 9.8 ± 2.2 pg/mL), followed by GA (9.1 ± 2.1 pg/mL) and GG (7.9 ± 2.0 pg/mL). Significant differences were found between GG and GA (*p* = 0.023) and between GG and AA (*p* = 0.004), with no difference between GA and AA.

For IL-10 −1082 G/A, no statistically significant differences in IL-10 levels were observed across genotypes (*p* = 0.26; Figure 3c), although a slight upward trend was noted from GG (4.9 ± 1.5 pg/mL) to GA (5.1 ± 1.6 pg/mL) and AA (5.3 ± 1.7 pg/mL).

In the case of IL1B -511 C/T, IL-1*β* expression differed significantly between genotypes (*p* = 0.003; Figure 3d). TT carriers exhibited the highest mean levels (8.5 ± 2.1 pg/mL), followed by CT (8.0 ± 2.0 pg/mL) and CC (7.2 ± 1.9 pg/mL), indicating a possible dose effect of the T allele.

Lastly, for IFN-*γ* +874 A/T, IFN-*γ* levels were similar across genotypes (*p* = 0.41; Figure 3e), with comparable mean concentrations among AA (5.1 ± 1.5 pg/mL), AT (5.3 ± 1.6 pg/mL), and TT (5.2 ± 1.7 pg/mL) individuals.

## 4. Discussion

In 2002, it was discovered that in patients with diabetes complicated by arthritis, sodium salicylate not only reduced inflammation and alleviated symptoms such as redness, swelling, heat, and pain caused by arthritis, but also lowered blood sugar levels [10], This finding drew attention to the role of inflammatory factors in diabetes, spurring a significant increase in research on the topic. In an obese mouse model, the severity of insulin resistance and diabetes increases with obesity, accompanied by significant alterations in inflammatory and anti-inflammatory factors. This highlights the strong correlation between inflammatory cytokines and the development of diabetes in the context of obesity [11, 12]. TNF-*α*, one of the most common inflammatory factors, has been closely linked to insulin resistance in animal models [13]. Consequently, many scholars believe that inflammation in adipose tissue is closely associated with insulin sensitivity. The development of gene knockout technology has further validated this hypothesis. In mouse models with knockouts of TNF-*α*, MCP-1, and toll-like receptors, free fatty acid levels were significantly reduced, and peripheral insulin sensitivity was enhanced [14, 15]. Furthermore, knocking out key factors in inflammatory cytokine signaling pathways, such as leukotriene receptor-1 and the cytotoxic T cell surface marker CD8, also led to increased insulin sensitivity [16, 17]. Among anti-inflammatory factors, IL-10 plays a role in suppressing the immune response by inhibiting the release of inflammatory mediators from Th1 cells, potentially providing a protective effect in diabetes. IL-1 receptor antagonists are another common group of anti-inflammatory factors; they inhibit other inflammatory cytokines, particularly members of the IL-1 family, and enhance the ability of pancreatic islets to secrete insulin. These antagonists are found at elevated levels in diabetic patients and increase further after the administration of hypoglycemic drugs, suggesting a possible protective role in diabetes [18, 19].

The incidence of GDM is steadily rising, not only imposing a significant economic burden on families and society but also potentially undermining a nation's long-term economic strength and cultural influence. Although some studies have begun to examine the specific changes in inflammatory factors across different BMI categories throughout pregnancy, the precise role of these factors in GDM remains unclear. Additionally, many studies are conducted in diverse populations, which introduce the possibility of population heterogeneity.

In our study, ROC curve analysis demonstrated that several inflammatory cytokines showed moderate diagnostic value in distinguishing GDM patients from healthy pregnant women. Notably, IL-6 exhibited the highest AUC (0.83), with satisfactory sensitivity and specificity, suggesting its potential as a useful biomarker for early GDM screening. Although IL-10 and TNF-*α* also showed promising AUC values, further validation in larger, prospective cohorts is needed. These findings support the hypothesis that inflammation-related markers may serve as non-invasive tools in GDM risk stratification, particularly when integrated with clinical parameters.

Most studies have shown that the concentrations of inflammatory factors in GDM patients are generally higher than those in healthy pregnant women [20–22]. However, studies from Brazil, Inner Mongolia, Denmark, Poland, and other regions have also reached conclusions similar to ours [23–25], showing no significant differences between GDM patients and normal pregnant women. Existing studies on inflammatory factors are conducted in diverse populations from different countries and regions, each with unique genetic backgrounds, dietary habits, and lifestyles. This leads us to hypothesize that the variations in inflammatory factor levels across populations may be due to the combined effects of diet, lifestyle, and genetics. Our findings demonstrate that specific cytokine gene polymorphisms are associated with altered plasma cytokine levels in women with GDM. The IL-6 −174 G/C and TNF-*α* −308 G/A variants were significantly associated with increased IL-6 and TNF-*α* expressions, respectively, suggesting that these promoter polymorphisms may enhance transcriptional activity and contribute to the heightened inflammatory state observed in GDM. Similarly, elevated IL-1*β* levels in TT carriers of the IL-1*β* −511 C/T polymorphism support a functional impact of this SNP on cytokine regulation. These genotype–expression correlations reinforce the hypothesis that genetic predisposition modulates inflammatory responses in GDM. In contrast, the lack of association for IL-10 −1082 G/A and IFN-*γ* +874 A/T polymorphisms may reflect compensatory mechanisms, tissue-specific regulation, or limited functional relevance in this context. Taken together, our results highlight the potential regulatory role of inflammatory gene variants in shaping the immunological environment of GDM and may inform future risk stratification or therapeutic targeting strategies. It should be noted that the control group in this study was in accordance with the HWE, which is of great significance for the reliability of the current research results. This law reveals the genetic laws governing the frequencies of genes and genotypes in a population, enabling the genetic performance of the population to remain relatively stable. Moreover, it also reveals the relationship between gene frequencies and genotype frequencies in a randomly mating population, thereby providing a method for calculating the gene frequencies and genotype frequencies of different populations in different situations, making genetics more predictable.

Regarding genetic polymorphisms, our study is the first to show that the IFN-*γ* +874 A/T polymorphism increases the risk of GDM. The IL-10 −1082 G/A polymorphism has also been linked to an elevated risk of GDM, as reported in several previous studies, though their conclusions differ [26, 27]. Even though these studies were conducted on Chinese populations, they involved different regions, which support our hypothesis of population heterogeneity affecting study outcomes. Meanwhile, we conducted a statistical analysis of gene expression and genotype. However, we found no correlation between the two. We suspect that the factors affecting gene expression are multifaceted and very complex. Through consulting relevant data, we found that gene expression levels are affected by a variety of factors, mainly including genetic factors, epigenetic factors, environmental factors, developmental stages and cell types, chromosome structure and location effects, and interaction networks. (1) Genetic factors: gene mutation and polymorphism are important factors affecting gene expression levels. Gene mutations include point mutations, insertions, or deletions that may result in changes in the level of gene expression. Gene polymorphism refers to the sequence differences of the same gene in different individuals, which may affect the expression and function of the gene. (2) Epigenetic factors: including DNA methylation, histone modification, and noncoding RNA. DNA methylation usually results in gene silencing by adding methyl groups to DNA molecules. Chemical modifications such as acetylation and methylation of histones can change the structure of chromatin and affect the accessibility and expression of genes. (3) Environmental factors, temperature, light, chemical substances, nutritional status, and so forth, can affect gene expression levels. Developmental stages and cell types: At different stages of development, different genes are activated or suppressed to direct cell differentiation and organ formation. Different types of cells have different patterns of gene expression that determine their specific functions and properties. (5) Effects of chromosome structure and location: Variations in chromosome structure such as inversion, translocation, and duplication may alter gene interactions and expression patterns. (6) Interaction networks: including gene–gene interaction and protein–protein interaction. There may be complex interactions between multiple genes that jointly regulate specific physiological processes. The products of gene expression may also interact with each other to form a complex regulatory network.

Compared with studies in other regions of China, the findings of this study (in Hebei) on the inflammatory status of pregnant women with GDM have both similarities and differences. First, many domestic studies have supported the existence of a systemic low-grade inflammatory state in pregnant women with GDM. The levels of IL-6, TNF-*α*, and IL-1*β* detected in this study were higher than those of normal pregnant controls, which is consistent with the results reported in Hangzhou, Zhejiang and other places: proinflammatory cytokines such as IL-6 and TNF-*α* were significantly increased in the GDM group. Similar phenomena were also observed in studies of the population in Inner Mongolia, North China. The levels of IL-6, IL-8, IL-1*β*, TNF-*α*, and other inflammatory factors in the serum and placenta of pregnant women with GDM were higher than those of healthy pregnant women, and the difference between the control group and the GDM group was significant. These consistencies indicate that the increase of inflammatory factors is one of the common characteristics of GDM, whether in the northern or southern population, and support the association of the pathogenesis of GDM with chronic inflammatory response. In contrast, the level of IFN-*γ* in this study did not change significantly (consistent with existing reports), and some studies even found that peripheral blood IFN-*γ* in patients with GDM was lower than that in normal pregnant women. This suggests that GDM-related immune disorders are mainly manifested as activation of innate immunity/proinflammatory factors, while changes in Th1 cytokines (such as IFN-*γ*) may not be as obvious as other factors. On the other hand, the changes in the anti-inflammatory factor IL-10 are not consistent in different regions: Hangzhou and other places found that the IL-10 level in the GDM group was lower than that in the control group (suggesting weakened anti-inflammatory regulation), but there are also studies (including Taiwan) reporting that the peripheral blood IL-10 of pregnant women with GDM is increased. Some scholars have proposed that the increase in IL-10 may be a compensatory response to inflammation, but this difference may also be related to the different gestational age, sample source and population characteristics. Therefore, the results of IL-10 expression in the GDM population in Hebei need to be interpreted with caution in the specific context. Overall, the results of this study on the increase in pro-inflammatory factors are reasonable in the Chinese context, but there are also limitations; that is, the baseline levels of inflammatory factors and immune responses in populations from different regions may be different, and the influence of regional and population-specific factors should be considered.

The analysis of inflammatory factor gene polymorphisms and GDM susceptibility in this study also needs to be discussed in the genetic context of the Chinese population. Due to racial/population differences, some functional sites that are often reported in European and American populations have extremely low frequencies in the Chinese population. For example, the −174 G/C polymorphism of the IL-6 promoter is extremely rare in the Han population (the vast majority of Chinese carry the −174 G allele), so domestic research has focused on other sites of the IL-6 gene (such as −572 C/G, rs1800796). The IL-6 gene polymorphism selected in this study is representative of the Chinese population. A study of the Hangzhou population has confirmed that the frequency of the G allele at the −572 site of the IL-6 gene is increased in GDM, and carrying the G allele can significantly increase the risk of GDM (OR≈1.4) [54]. Our results are consistent with this, indicating that IL-6 gene variation may also promote high expression of IL-6 and insulin resistance in the Hebei population. For the IL-10 gene, most studies in the Chinese population have focused on its common promoter sites (such as −1082 A/G, rs1800896). Data from the Hangzhou population showed that IL-10 gene polymorphism was associated with GDM (the risk of GDM in carriers of a certain allele increased by nearly three times), but in studies in Taiwan and other regions, IL-10 promoter site polymorphism did not show a significant association. The results of IL-10 gene polymorphism detected in our Hebei samples did not fully replicate all the findings in other places. The possible reason is that there are differences in IL-10 allele frequencies among people in different regions, or the sample size is not enough to observe a weak effect. This can also be confirmed by the new findings of Taiwanese scholars: Compared with traditional sites, a previously unreported IL-10 gene SNP (rs3021094, located in the intron region of the gene) showed a stronger association in Chinese GDM, and carriers of mutant alleles had elevated plasma IL-10 and an increased risk of GDM. This result suggests that different populations may be affected by different gene variants, and the fact that this study did not examine some new sites may be a major limitation. In addition, regional differences in TNF-*α* gene polymorphism are also worth noting. The TNF-*α* we detected the −308 G/A polymorphism showed an association trend with the risk of GDM in the Hebei population, which is consistent with the results reported in the Hangzhou population. In the study of the Inner Mongolia population, the investigators did not find the effect of the −308 site, but reported that another site, TNF-*α*-857 C/T, was significantly associated with GDM. This difference may be due to the different allele distributions among different ethnic groups, or due to differences in sample size and study design. In view of this, the conclusions of this study on the relationship between gene polymorphism and GDM susceptibility are mainly applicable to the Han population in Hebei, and the regional and ethnic background should be carefully considered when extending to other regions. For example, differences in lifestyle between ethnic minority areas or coastal and inland areas may affect the effects of inflammation-related genes. In summary, the rationality of this study is that most of the findings are consistent with the existing research trends in China, supporting the inflammatory pathogenesis of GDM; but the limitation is that the differences in genetic background and environmental factors between regions may lead to different inflammatory indicators and gene effects, and more multicenter studies are needed to verify our conclusions in Hebei.

## 5. Limitation

This study has the following limitations that need to be noted. First, the family history of diabetes of the participants was not collected, so it could not be included in the analysis as a potential confounder. GDM has a clear genetic susceptibility, and women with diabetes in the family have a significantly increased risk of GDM. If not controlled, the strength of the association between certain inflammatory factors or gene polymorphisms and the risk of GDM may be overestimated. Therefore, future studies should systematically collect family history information and perform stratification or correction in the analysis to improve the reliability of causal inference. Second, this study lacks data on gestational order (pregnancy order) and fails to distinguish the occurrence of GDM between first pregnancy and multiple pregnancies. Previous studies have shown that the risk of GDM increases with the number of pregnancies, which may be partly related to the accumulation of insulin resistance and limited metabolic adaptation. Therefore, the failure to consider the variable of gestational order may affect the interpretation and extrapolation of the results. Future studies should systematically include gestational order data, combine reproductive history with metabolic indicators, and further explore its regulatory effect on the risk of GDM through interaction with inflammatory factor expression and gene variation. Future multicenter, prospective studies should pay more attention to the completeness of individual basic information, especially the control of family history and reproductive history variables, to achieve more comprehensive and accurate risk assessment and mechanism exploration. Lastly, bioinformatics and meta-analysis are emerging disciplines that emerged with the launch of the HGP, which integrates mathematics, computer science and biology to elucidate the biological significance of various types of data. Currently, bioinformatics and meta-analysis play a pivotal role in the development of medicine [28–41]. The current research has not linked bioinformatics or meta-analysis with GDM.

## 6. Conclusion

In summary, elevated plasma levels of IL-1, IL-6, IL-10, and TNF-*α* were observed in GDM patients. Additionally, polymorphisms such as IL-1*β* −511 C/T, IL-6 −174 G/C, IL-10 −1082 G/A, IFN-*γ* +874 A/T, and TNF-*α* −308 G/A showed a strong correlation with increased GDM risk in the Han women from northern China (specifically, Hebei Province). Pregnant women with ACC haplotypes of IL-10 have a lower risk of GDM. Cytokine gene polymorphisms in IL6, TNFA, and IL-1B are associated with altered inflammatory profiles in GDM, suggesting a genetic contribution to disease-related immune dysregulation. Our study suggests that these factors have the potential to serve as biomarkers for the diagnosis and clinical prognosis of GDM in Han women from northern China (Hebei Province).

## Figures and Tables

**Table 1:** The participants' characteristics of both the GDM group and control group.

**Basic information**	**Control (** **N** = 280**)**	**GDM group (** **N** = 160**)**	**p**
Age (years)	30.17 ± 3.80	31.41 ± 4.50	0.053
TG (mmol/L)	2.10 ± 0.64	2.25 ± 0.80	0.156
TC (mmol/L)	6.04 ± 1.00	5.75 ± 1.10	0.073
LDL-C (mmol/L)	3.39 ± 0.78	3.24 ± 0.86	0.225
HDL-C (mmol/L)	2.00 ± 0.33	1.90 ± 0.34	0.053

Abbreviation: GDM, gestational diabetes mellitus.

**Table 2:** IL-1B genotype and allele frequency for patients with GDM.

**IL-1B loci**	**Control group (** **N** = 280**)**		**GDM group (** **N** = 160**)**		**OR (95% CI)** ^**a**^	*p* ^**a**^
	**n**	**Percentage (%)**	**n**	**Percentage (%)**		
−511 C/T						
CC	69	24.6	20	10.0	1.00^REF^	
TC	71	25.4	44	27.5	**2.12 (1.37–3.30)**	**< 0.001**
TT	140	50.0	96	62.5	**2.34 (1.57–3.48)**	**< 0.001**
C	209	37.3	84	23.6	1.00^REF^	
T	351	62.7	236	76.4	**1.66 (1.34–2.06)**	**< 0.001**
+3954 C/T						
CC	146	52.1	82	51.2	1.00^REF^	
TC	112	40.0	64	40.0	1.02 (0.76–1.36)	0.907
TT	22	7.9	14	8.8	1.13 (0.68–1.89)	0.632
C	404	72.1	228	71.3	1.00^REF^	
T	156	27.9	92	28.7	1.04 (0.84–1.30)	0.689

*Note:* The bolded data are significant in terms of statistics. “REF” represents “reference group.”

Abbreviations: CI, confidence interval; GDM, gestational diabetes mellitus; OR, odds ratio.

^a^Adjusted for sex and age by logistic regression model.

**Table 3:** IL-6 genotype and allele frequency for patients with GDM.

**IL-6 loci**	**Control group (** **N** = 280**)**		**GDM group (** **N** = 160**)**		**OR (95% CI)** ^**a**^	*p* ^**a**^
	**n**	**Percentage (%)**	**n**	**Percentage (%)**		
−174 G/C						
GG	99	35.4	34	21.3	1.00^REF^	
GC	114	40.7	82	51.2	2.09 (1.19–2.95)	**< 0.001**
CC	67	23.9	44	27.5	2.91 (1.31–2.81)	**< 0.001**
G	312	55.7	150	46.9	1.00^REF^	
C	248	44.3	170	53.1	1.43 (1.17–1.73)	**< 0.001**
−1363 G/T						
GG	151	53.9	89	55.6	1.00^REF^	
GT	112	40.0	60	37.5	1.91 (0.60–1.37)	0.647
TT	17	6.1	11	6.9	1.10 (0.49–2.45)	0.820
G	302	73.9	238	74.4	1.00^REF^	
T	146	26.1	82	25.6	1.98 (0.71–1.34)	0.884

*Note: *The bolded data are significant in terms of statistics. “REF” represents “reference group.”

Abbreviations: CI, confidence interval; GDM, gestational diabetes mellitus; OR, odds ratio.

^a^Adjusted for sex and age by logistic regression model.

**Table 4:** IL-10 genotype and allele frequency for patients with GDM.

**IL-10 loci**	**Control group (** **N** = 280**)**		**GDM group (** **N** = 160**)**		**OR (95% CI)** ^**a**^	*p* ^**a**^
	**n**	**Percentage (%)**	**n**	**Percentage (%)**		
−592 C/A						
CC	78	27.9	38	23.8	1.00^REF^	
CA	112	40.0	76	47.5	1.39 (0.86–2.37)	0.180
AA	90	32.1	46	28.7	0.91 (0.53–1.56)	0.738
C	268	47.9	152	47.5	1.00^REF^	
A	292	52.1	118	52.5	1.01 (0.77–1.34)	0.919
−1082 G/A						
GG	102	36.4	40	25.0	1.00^REF^	
GA	114	40.7	77	48.1	1.72 (1.24–2.39)	**0.001**
AA	64	22.9	43	26.9	1.71 (1.18–2.50)	**0.005**
G	318	56.8	157	49.1	1.00^REF^	
A	242	43.2	163	50.9	1.36 (1.12–2.66)	**0.002**
−819 T/C						
TT	162	57.8	100	62.5	1.00^REF^	
TC	110	39.3	54	33.8	1.80 (0.45–1.42)	0.530
CC	8	2.9	6	3.7	1.22 (0.26–5.66)	0.881
T	434	77.5	254	79.4	1.00^REF^	
C	126	22.5	66	20.6	1.90 (0.56–1.44)	0.735

*Note:* The bolded data are significant in terms of statistics. “REF” represents “reference group.”

Abbreviations: CI, confidential index; GDM, gestational diabetes mellitus; OR, odds ratio.

^a^Adjusted for sex and age by logistic regression model.

**Table 5:** IL-17 genotype and allele frequency for patients with GDM.

**IL-17 loci**	**Control group (** **N** = 280**)**		**GDM group (** **N** = 160**)**		**OR (95% CI)** ^**a**^	*p* ^**a**^
	**n**	**Percentage (%)**	**n**	**Percentage (%)**		
rs2275913						
GG	145	51.8	80	50.0	1.00^REF^	
GA	112	40.0	66	41.3	1.07 (0.71–1.61)	0.752
AA	23	8.2	14	8.7	1.10 (1.54–2.26)	0.789
G	402	71.8	226	70.6	1.00^REF^	
A	158	28.2	94	29.4	1.06 (0.78–1.43)	0.714
rs763780						
TT	151	53.9	89	55.6	1.00^REF^	
TC	112	40.0	60	37.5	1.91 (0.60–1.37)	0.647
CC	17	6.1	11	6.9	1.10 (0.49–2.45)	0.820
T	414	73.9	238	74.4	1.00^REF^	
C	146	26.1	82	25.6	1.98 (0.71–1.34)	0.884

Abbreviations: CI, confidence interval; GDM, gestational diabetes mellitus; OR, odds ratio.

^a^Adjusted for sex and age by logistic regression model.

**Table 6:** IFN-*γ* genotype and allele frequency for patients with GDM.

**IFN-*γ* loci**	**Control group (** **N** = 280**)**		**GDM group (** **N** = 160**)**		**OR (95% CI)** ^**a**^	*p* ^**a**^
	**n**	**Percentage (%)**	**n**	**Percentage (%)**		
+874 A/T						
AA	151	53.9	79	49.4	1.00^REF^	
AT	112	40.0	60	37.5	1.02 (0.68–1.55)	0.911
TT	17	6.1	21	13.1	**2.36 (1.18–4.73)**	**0.014**
A	414	73.9	218	68.1	1.00^REF^	
T	146	26.1	102	31.9	1.33 (0.98–1.79)	0.066
+2108 A/G						
AA	143	51.1	80	50.0	1.00^REF^	
AG	112	40.0	66	41.3	1.05 (0.70–1.59)	0.803
GG	25	8.9	14	8.7	1.00 (0.49–2.03)	0.998
A	398	71.1	226	70.6	1.00^REF^	
G	162	28.9	94	29.4	1.02 (0.76–1.38)	0.888

*Note:* The bolded data are significant in terms of statistics. “REF” represents “reference group.”

Abbreviations: CI, confidence interval; GDM, gestational diabetes mellitus; OR, odds ratio.

^a^Adjusted for sex and age by logistic regression model.

**Table 7:** TNF-*α* genotype and allele frequency for patients with GDM.

**TNF-*α* loci**	**Control group (** **N** = 280**)**		**GDM group (** **N** = 160**)**		**OR (95% CI)** ^**a**^	*p* ^**a**^
	**n**	**Percentage (%)**	**n**	**Percentage (%)**		
−308 G/A						
GG	101	36.1	38	23.8	1.00^REF^	
GA	114	40.7	79	49.4	1.84 (1.05-2.95)	**0.011**
AA	65	23.2	43	26.8	1.76 (1.03-3.01)	**0.038**
G	316	56.4	155	48.4	1.00^REF^	
A	244	43.6	165	51.6	1.65 (1.25-2.17)	**<0.001**
−863 C/A						
CC	152	54.3	90	56.3	1.00^REF^	
CA	112	40.0	60	37.5	1.90 (0.51-1.61)	0.847
AA	16	5.7	10	6.2	1.06 (0.33-3.42)	0.832
C	416	74.3	240	75.0	1.00^REF^	
A	144	25.7	80	25.0	1.96 (0.62-1.51)	0.959

*Note:* The bolded data are significant in terms of statistics. “REF” represents “reference group.”

Abbreviations: CI, confidence interval; GDM, gestational diabetes mellitus; OR, odds ratio.

^a^Adjusted for sex and age by logistic regression model.

**Table 8:** The individual IL-10 haplotype carrier frequency in the GDM and control groups.

**Haplotype frequency**	**Control (** **N** = 280**)**	**GDM group (** **N** = 160**)**	**p**
ATA	0.65	0.71	0.14
ACC	0.27	0.18	0.03
GCC	0.08	0.11	0.25

Abbreviation: GDM, gestational diabetes mellitus.

## Data Availability

All data generated or analyzed during this study is included in this published article.
